# Opportunities, Challenges, and Perspectives for Equity and Capacity Building in Robotic Urological Surgery in Africa

**DOI:** 10.1590/S1677-5538.IBJU.2025.0356

**Published:** 2026-06-03

**Authors:** Abdelkerim Saleh Nedjim, Ouima Justin Dieudonné Ziba, Kaleab Habtemichael Gebreselassie, Abdullahi Khalid, Marcella D. C. Biyouma, Anteneh Tadesse Kifle

**Affiliations:** 1 Adam Barka University Abeche Chad Adam Barka University, Abeche, Chad; 2 Modern Urology for Africa Casablanca Morroco Modern Urology for Africa (MUFA), Casablanca, Morroco; 3 Yalgado Ouédraogo Teaching Hospital Ouagadougou Burkina Faso Yalgado Ouédraogo Teaching Hospital, Ouagadougou, Burkina Faso; 4 Worabe Comprehensive Specialized Hospital Worabe Ethiopia Worabe Comprehensive Specialized Hospital, Worabe, Ethiopia; 5 Usmanu Danfodiyo University and Teaching Hospital Sokoto Nigeria Usmanu Danfodiyo University and Teaching Hospital, Sokoto, Nigeria; 6 Douala Laquintinie Hospital Douala Cameroon Douala Laquintinie Hospital, Douala, Cameroon; 7 P.C.E.A Chogoria Hospital Chogoria Kenya P.C.E.A Chogoria Hospital, Chogoria, Kenya

To the editor,

We read with great interest the article by Moschovas et al. (1) entitled "Redefining Surgical Collaboration: The First Structured Transcontinental Robotic Telementoring Experience Between Africa and South America." This study emerges within the context of the rapid evolution of robotic surgery, now widely recognized as a major advancement in minimally invasive surgery, improving technical precision, three-dimensional visualization, and postoperative outcomes while reducing tissue trauma and surgical complications (2). Robotic surgery is currently implemented across several surgical specialties through a variety of technological platforms (3). Concurrently, telementoring and telesurgery have emerged as innovative tools capable of delivering surgical expertise remotely and reducing disparities in access to specialized care (4). These approaches are particularly relevant in low- and middle-income countries, where shortages of skilled personnel and infrastructural limitations continue to hinder the adoption of advanced surgical technologies (5).

Moschovas et al. (1) highlighted the feasibility and safety of a structured transcontinental robotic telementoring model. This achievement builds upon the earliest proof-of-concept experiences in remote robotic surgery and demonstrates that international surgical collaboration can be conducted safely when supported by a clear distribution of responsibilities, an autonomous local surgical team, and rigorous ethical oversight (6). Previous studies have already shown that telesurgery and telementoring may improve access to surgical care in resource-limited regions while simultaneously promoting knowledge transfer and standardization of surgical practices (5). Within the African context, transcontinental robotic telementoring represents a major strategic opportunity to strengthen local capacities in minimally invasive and robotic surgery by enabling real-time interaction between African centers and international expert teams, providing indirect access to highly specialized expertise without requiring the physical displacement of either patients or surgeons (7).

Recent evidence has further reinforced the technical feasibility and reproducibility of long-distance robotic telesurgery. In a pioneering study conducted between China and Kuwait over a distance of approximately 7,000 kilometers, Aldousari et al. (8) reported successful completion of robotic radical prostatectomies and partial nephrectomies without clinical or technical complications using a low-latency fiber-optic and 5G network infrastructure. Likewise, a recent systematic review by Ferreira et al. (9) analyzing 54 urological telesurgical procedures demonstrated that robotic telesurgery can be safely performed with acceptable operative outcomes, minimal blood loss, and no conversion to open surgery, despite the considerable geographic distances involved. Beyond the surgical team itself, the safe deployment of telesurgery also requires a fundamental reframing of anesthetic practice. As Barani et al. (10) recently emphasized, telesurgery represents a natural evolution of robotic surgery in which established anesthetic protocols, the mandatory presence of an on-site surgical and anesthetic team, and structured communication strategies are essential to ensure patient safety and optimize outcomes. Beyond these technical and clinical considerations, as recently demonstrated in the Brazilian experience, the safe implementation of telesurgery requires clear ethical and legal frameworks, rigorous research governance, and strong multidisciplinary collaboration involving surgical societies, hospitals, governmental agencies, and technology providers (11).

Despite these promising advances, the adoption of robotic telesurgery across Africa remains constrained by major structural, economic, and technological barriers. These include the high cost of robotic platforms, maintenance expenses, limited access to consumables, insufficient specialized training programs, and fragile hospital infrastructures characterized by recurrent power outages and unstable high-speed internet connectivity (7). The cybersecurity vulnerabilities inherent to remote surgical systems, the protection of medical data transmitted across international networks, the complexity of informed consent in transnational settings, and unresolved questions of medico-legal liability further compound these challenges (12). In this context, the progressive development of regional hubs, reinforcement of digital infrastructures, implementation of hybrid training models, and strengthening of South–South collaborations could facilitate sustainable knowledge transfer and contribute to reducing inequalities in access to specialized surgical care — ultimately making telesurgery a genuine instrument of equity across the African continent.

The Authors
